# Spleen Rupture in a Case of Untreated *Plasmodium vivax* Infection

**DOI:** 10.1371/journal.pntd.0001934

**Published:** 2012-12-13

**Authors:** André Machado Siqueira, Belisa Maria Lopes Magalhães, Gisely Cardoso Melo, Mireia Ferrer, Paola Castillo, Lorena Martin-Jaular, Carmen Fernandez-Becerra, Jaume Ordi, Antonio Martinez, Marcus Vinícius Guimarães Lacerda, Hernando A. del Portillo

**Affiliations:** 1 Fundação de Medicina Tropical Dr. Heitor Vieira Dourado (FMT-HVD), Manaus, Brazil; 2 Universidade do Estado do Amazonas, Manaus, Brazil; 3 Barcelona Centre for International Health Research (CRESIB, Hospital Clinic–Universitat de Barcelona), Barcelona, Spain; 4 Laboratory of Pathology, Barcelona, Spain; 5 Institució Catalana de Recerca I Estudis Avançats, Barcelona, Spain; Emory University, United States of America

## Introduction

We report the unique case of a 19-year-old nonimmune patient with *Plasmodium vivax* monoinfection, confirmed by PCR in the peripheral blood and in the spleen section, who was splenectomized due to spleen rupture two days prior to the diagnosis and treatment of the malarial infection. Microscopic analyses evidenced white pulp expansion and a diffuse hypercellularity in the splenic red pulp, with intense proliferating plasmablasts in the subcapsular and perivascular compartments as well as large numbers of intact *P. vivax*–infected reticulocytes in the cords, in the absence of other concomitant infectious diseases. To our knowledge, this is the first full detailed immunohistopathological characterization of a nontreated *P. vivax*–infected spleen.

## Description of Case

A 19-year-old man was admitted at a secondary-care hospital in the Brazilian Amazon with acute abdominal pain after jumping from a tree denying to have suffered any direct abdominal trauma. A diagnosis of spleen rupture and intra-abdominal haemorrhage was performed. He was submitted to splenectomy, during which a large spleen of 1,300 grams with a capsule rupture in the colic surface was obtained. The patient lived in an urban area of Manaus and denied previous episodes of malaria. For the last month before the traumatic event he had been working in a building project in a rural area where malaria transmission is endemic and referred that in the two days preceding surgery he had been feeling feverish and complaining of mild headache and asthenia. After surgery, he developed high fever, chills, and generalized myalgia. The initial diagnostic investigation performed by the Surgery Department only revealed anemia (Hb 8.7 g/dL) and thrombocytopenia (66,000 platelets/mm^3^). The patient was then transferred to a tertiary-care infectious diseases reference center.

A thick blood smear disclosed *P. vivax* infection with a semiquantitative parasitemia between 10.000 and 100.000 parasites/mm^3^. Real-time polymerase chain reaction (PCR) from the peripheral blood confirmed *P. vivax* monoinfection. Treatment was initiated with chloroquine (1,500 mg divided in 3 days) and primaquine (30 mg/day during 7 days). Both fever and parasitemia were cleared within 48 hours. The patient was discharged in the third day of treatment, and he did not develop any relapse nor had any other complications through a 1-year follow-up.

The study was approved by the Institutional Review Board of the Fundação de Medicina Tropical Heitor Vieira Dourado and the National Committee of Ethics in Science and Technology (CONEP Process No.: 25.001.011.792/2009-15), and the patient consented his case to be published.

## How Frequent Are Splenomegaly and Spleen Rupture in Malaria Infection? Are *P. vivax*–Infected Patients at Higher Risk of Complications?

Enlargement of the spleen is a well-known clinical feature of malaria, and it is estimated to occur in 70%–80% of acute cases, with its size normalizing after successful treatment [1]. In areas of intense transmission, the spleen is palpable in 50%–80% of individuals, being directly associated with immunity acquisition, as shown by its higher prevalence in children and correlation with both antibody levels and host genetics [2], [3]. More reliable and accurate diagnostics techniques have been used to measure malaria transmission, but for a long time, especially during WHO's Malaria Eradication Campaign in the 1950–60s, the proportion of palpable spleens in a given population (the spleen rate) was used to the deployment of control efforts, highlighting its epidemiological utility [4], [5].

Left-upper-quadrant abdominal pain in a patient with malaria should prompt the physician to consider the diagnosis of splenic infarction or spleen rupture [6], [7]. The real incidence of splenic complications is unknown as there is substantial underreporting of mild, asymptomatic, and nonsevere cases, but some reports point that it can be as high as 8.8% among patients with abdominal pain [8]. An extensive review of published cases of splenic rupture demonstrated that it is most commonly observed during the primary attack in nonimmune individuals [9]. Although there were no differences in the clinical presentation related to the infecting species, it seems that *P. vivax* leads to more marked spleen enlargement than infection with other species [9]. The pathophysiological events leading to rupture are not completely understood, but it seems that it is mainly a mechanical event related to the rapid enlargement of the organ with preceding splenic infarction occurring in a minority of cases [9].

## What Are the *P. vivax*–Induced Changes in the Spleen?

**Figure 1:**
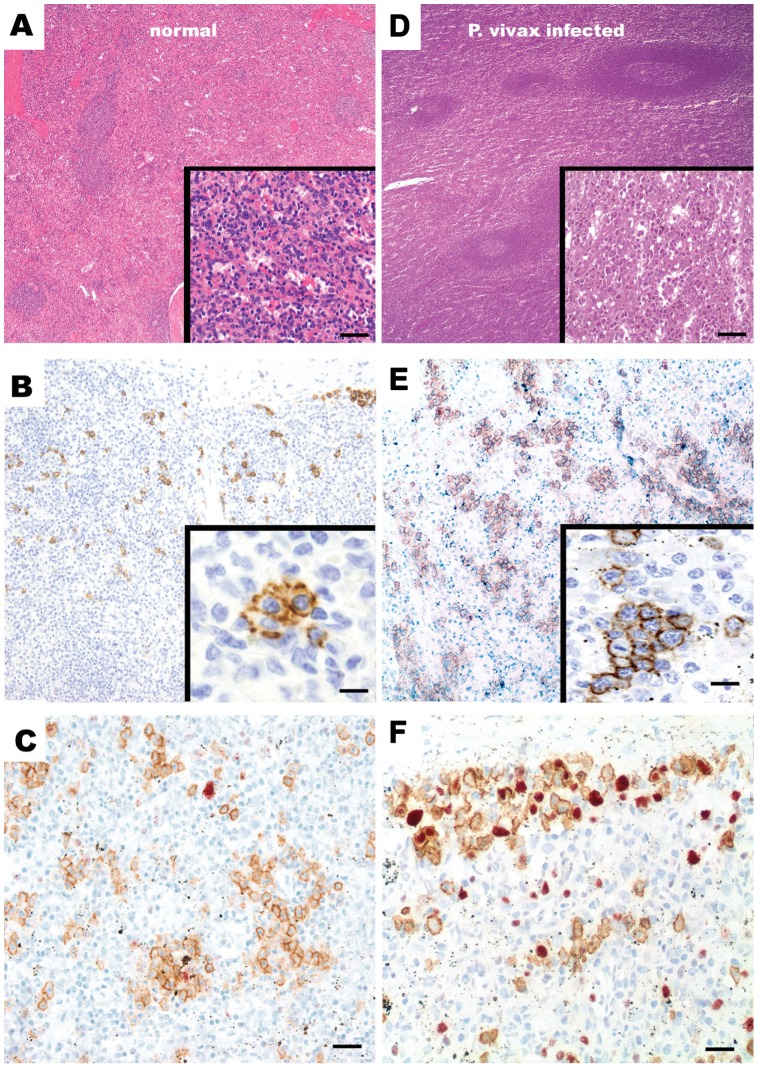
Immunohistochemical staining of *P. vivax*–infected and normal spleen sections. HE staining of the normal (A) and *P. vivax*–infected (B) spleen sections evidencing, respectively, periarteriolar lymphoid cuffs and red pulp cords depleted of lymphoid cells (inset 20× normal) and a prominent white pulp expansion with hyperplastic germinal centers in secondary lymphoid follicles highlighting in the inset a striking red cord infiltration by immunoblasts and reactive plasma cells (20× *P. vivax*–infected). (C) The normal spleen reveals a scattered CD138 positive plasma cell distribution in the subcapsular and perivascular compartments (2×); higher magnification view shown in the inset (40×). (D) In contrast, a marked increase of CD138 positive plasma cells is observed in the *P. vivax*–infected spleen (2×), including the detection of mitotic activity among several CD138 positive plasma cells shown in the inset (40×). (E) Double immunostaining with CD138 (brown) and Ki-67 (red) demonstrated the lack of proliferation in plasma cells in the normal spleen (20×). (F) In contrast, there is a significant increase in CD138 and Ki67 positive plasmablasts in the *P. vivax*–infected spleen (20×).

Histologically, hematoxylin-eosin (HE) staining revealed that, compared to a normal spleen (Figure 1A), the most significant changes in the *P. vivax*–infected spleen were a white pulp expansion and a diffuse hypercellularity in the splenic red pulp (Figure 1B). The periarteriolar lymphoid sheets of the white pulp were enlarged, and well-developed secondary lymphoid follicles were easily found. A prominent infiltration by immunoblasts and plasma cells was observed in the cords as well as a striking intrasinusoidal histiocytosis. Although the histological characteristics of the spleen resembled a diagnosis of B-cell lymphoma, it could be excluded after performing additional tests. A nested PCR performed in the spleen sections demonstrated the presence of *P. vivax* and excluded coinfection with *P. falciparum* (not shown). Infections by HIV, EBV, CMV, and HHV8 were not detected through immunohistochemistry and in situ hybridization. Thus, *P. vivax* infection was the only condition affecting the patient's spleen after extensive diagnostic investigation.

A series of cell markers was used to determine the effects of *P. vivax* infection in this organ (Table S1). Noticeably, a mild follicular hyperplasia (CD20, CD10), mild red cord hyperplasia (CD2, CD3, CD5, CD7), expansion of monocytes-macrophages (CD68), and plasmablast expansion and proliferation (CD138, MUM-1, Ki 67) in subcapsular and perivascular areas, as well as large expression of B-cell and antibody markers (IgM, IgG, IgD, Lambda, and Kappa light chains) were observed when compared to sections from the spleen of a normal individual who suffered a trauma-forcing splenectomy. In contrast, none of the other markers used to identify T cells (Granzyme B, CD4, CD8, CD57, FOXP3, and TCRbeta), dendritic cells (CD123), natural killer cells (CD56), NK cells and histiocytes (CD16), endothelial cells (CD31 and CD34), myeloid and monocytic cells (CD33), neutrophil granulocytes/monocytes (myeloperoxidase), normal erythroid cells at all stages of differentiation (glycophorin A), or megakaryocytes (CD61) showed a difference in location or expression of these receptors in the spleen from the *P. vivax* patient as compared to the normal spleen (Table S1). Worth highlighting, as compared to the normal spleen (Figure 1C), a marked increase in CD138 positive plasma cells was observed in the *P. vivax–*infected spleen (Figure 1D). Moreover, only very rare CD138 positive cells were in mitosis in the normal spleen (Figure 1C, inset), whereas abundant mitotic figures were found in the *P. vivax*–infected spleen (Figure 1D, inset). A double immunostaining with CD138 and the Ki-67 proliferation marker confirmed the plasmablastic expansion of double Ki-67 and CD138 positive cells in the *P. vivax*–infected spleen in the subcapsular and perivascular compartments (Figure 1E and 1F).

**Figure 2:**
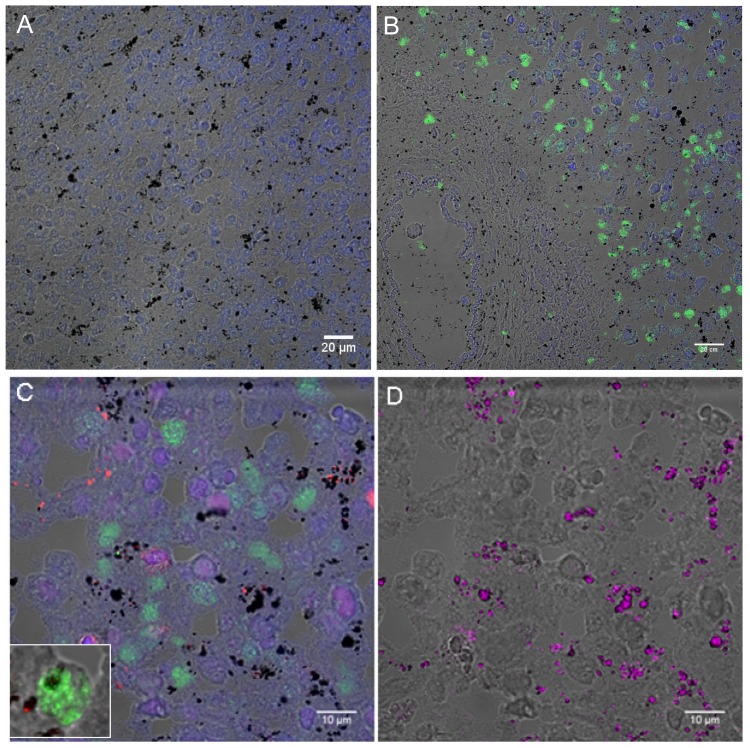
Immunohistofluorescence images of *P. vivax*–infected spleen sections. (A) Spleen section showing *P. vivax* parasites mostly in the cords of the red pulp as detected by polyclonal antibodies against Vir proteins. (B) Negative control using preimmune sera. Nuclei are shown in blue and the bright field image of the tissue in gray. Scale bar: 20 µm. (C) Double staining showing CD68 macrophages in red and parasites stained in green. Scale bar is 10 µm. (D) Reflection contrast (magenta) was used to detect parasite pigment.

Immunofluorescence assays of spleen sections were performed using antibodies raised against conserved motifs of *P. vivax* VIR proteins, previously shown to specifically recognize *P. vivax*–infected reticulocytes from human patients [10], to determine the presence of parasites. Results demonstrated the presence of large numbers of parasites in the red pulp (Figure 2A) and specificity was demonstrated using pre-immune sera (Figure 2B). Noticeably, confocal images using anti–*P. vivax* VIR and anti-CD68 (a marker of macrophages) antibodies revealed intact *P. vivax*–infected reticulocytes characterized by dotted pattern of staining mostly outside macrophages (Figure 2C and inset) and the presence of large amounts of parasite pigment as revealed by reflection contrast (Figure 2D).

## Discussion

Here, a 19-year-old man suffered a traumatic spleen rupture in the course of an acute untreated nonsevere infection with *P. vivax* and was submitted to splenectomy due to profuse intra-abdominal hemorrhage. In a malaria-endemic tropical setting such as the Western Brazilian Amazon region, a thick blood smear must be included in the initial diagnostic work-up, as it can reliably provide a prompt diagnosis and treatment. This unique case allowed us to determine the histopathological effects of an active *P. vivax* infection in the spleen of a nonimmune individual. Our findings revealed that in addition to well-described splenomegaly and diffuse cellular hyperplasia associated with malaria infections, there were intense proliferating plasmablasts in the subcapsular and perivascular compartments as well as intact *P. vivax*–infected reticulocytes outside macrophages in the cords.

The spleen is a complex organ involved in both the removal of damaged and parasitized red blood cells and in the generation of immunity, consequently having a pivotal role in malaria [11], [12]. In *P. falciparum*, several structural and functional changes have been comprehensively described, and these have been associated with the capacity of parasitized cells clearance and severe disease manifestations. The essential role of the spleen in the control of parasite loads is also reinforced by higher parasitemias and increased clinical severity in patients who suffered splenectomy [13]. Histopathological analyses of the spleen in natural malaria infections have been limited to snapshots of postmortem specimens in patients mostly having received antimalarial therapy; our patient's misfortune provided a rare opportunity to explore extensively an untreated *P. vivax*–infected spleen.

In an extensive study of the spleen from Vietnamese patients who died from late complications of *P. falciparum* infection, attention was drawn to a marked architectural disorganization and loss of B cells from the marginal zone [14]. However, as the authors themselves emphasize, these alterations are very unlikely to reflect what occurs in the majority of patients who are able to control the infection. The review of spleen rupture from *P. vivax–*infected patients published by Lubitz in 1949 remains the most complete description of the alterations in the acute infection by this parasite so far [15]. In this study, most cases had acute infection and showed follicle hyperplasia with active germinal centers and stretching of splenic parenchyma and capsule. Similar observations were made in two other cases of spleen rupture due to *P. vivax* [16]. Our results also showed white pulp expansion and a diffuse hypercellularity. Moreover, the use of different cellular markers allowed us to identify an increase in B cells, plasmablasts and plasma cells, all of them implicated in humoral antibody responses. Although intense proliferation of B cells, resembling splenic commitment of the spleen had been previously described in two hyperreactive malarial splenomegaly (HMS) case [17], this is the first report showing these similar histopathological features in an acute *P. vivax* infection, highlighting how difficult it can be to distinguish between malarial infection and malignant disorders in endemic settings.

B cells and antibodies play a prominent role in the development of immunity against asexual infections in human malaria [18], [19]. Fast production of antibodies is elicited by B cells, which after antigen encounter migrate to T-cell-rich zones of secondary lymphoid organs, including the spleen. From there, independently of whether B cells encounter T-cell-dependent or T-cell-independent antigens, B cells become plasmablasts for subsequent differentiation of plasma cells [20]. Noticeably, in experimental infections of C56BL/6 mice with the *P. chabaudi chabaudi* AS nonlethal strain, remodeling of the spleen and induction of plasmablasts in extrafollicular compartments have been described [21]. Similar results have been obtained in experimental infections of Balb/c mice with the *P. yoelii* 17× reticulocyte-prone nonlethal strain (unpublished data). Interestingly, a population of extrafollicular splenic plasmablasts responsible for T-cell-independent antibody responses has also been described in mice infected with the intracellular bacterial pathogen *Erlichia muris* [22]. In humans, this infection causes a disease known as ehrlichioses, which is characterized by splenomegaly, lymphopenia, and thrombocytopenia, thus resembling the clinical symptoms of the *P. vivax* patient reported here. In striking contrast, no extrafollicular plasmablast proliferation was observed in histopathological examinations of the spleen of patients dying of severe falciparum malaria. It is thus tempting to speculate that *P. vivax* infections elicit extrafollicular plasmablastic proliferation and a large T-cell-independent immune response.

In spite of an intense B-cell antibody plasmablastic response and expanded number of intrasinusoidal macrophages in the cords, immunofluorescence analysis revealed the presence of large numbers of *P. vivax*–infected reticulocytes in the cords. Although it is plausible that some of these parasites were simply detected in their passage through the spleen, it is difficult to reconcile this sole explanation with the large numbers detected and the co-localization studies demonstrating that they were mostly outside macrophages. Noticeably, cytoadherence of the reticulocyte-prone nonlethal *P. yoelii* 17× strain to a spleen blood barrier of fibroblastic origin has been shown [23]. Most important, cytoadherence of *P. vivax*–infected reticulocytes to different endothelial receptors have been recently demonstrated [24], [25]. Whether the large numbers of *P. vivax*–infected reticulocytes observed in the cords are due to mechanical trapping [13] as opposed to active adherence remains to be determined.

## Conclusion

In endemic areas, malaria is a main cause of spleen rupture and should always be considered in the initial work-up. Although most cases of spleen rupture can be managed conservatively, surgery may be necessary when severe hemorrhage occurs. The misfortune of our patient provided the unique opportunity of performing, to the best of our knowledge, the first detailed immunohistopathological study of a human spleen from a *P. vivax* patient having an active infection with no other clinical confounding effects and that had not been drug-treated before splenectomy. Similar to *P. falciparum*, *P. vivax* infections induced white pulp expansion and a diffuse hypercellularity in the splenic red pulp. In contrast to *P. falciparum*, *P. vivax* induced a striking proliferation of plasmablasts in extrafollicular compartments of the spleen. This characteristic of the spleen resembled a diagnosis of B-cell lymphoma leading to an extensive work-up to clarify the patient's diagnosis. Last, our observations add to experimental and histopathological evidence [10], [24], [26], [27], challenging the paradigm that there is no sequestration in deep microvasculature in *P. vivax* infections. The presence of large numbers of intact *P. vivax*–infected reticulocytes observed in the cords is the matter of further investigation.

**Key Learning Points**

- *Plasmodium* infection is an important cause of atraumatic spleen rupture in malaria-endemic areas.
- *P. vivax* infection leads to several macroscopic and microscopic changes in the infected spleen.
- The splenic microscopic changes induced by malarial infection can resemble lymphoma and an extensive differential workout might be necessary.
- The massive proliferation of plasmablasts in the *P. vivax*–infected spleen and the large numbers of intact *P. vivax*–infected reticulocytes observed in the cords may have important implications on the acquisition of innate immunity in vivax malaria.

## Supporting Information

Table S1 Markers, application, and findings used in the phenotypic in situ characterization and distribution of cells in the spleen of the *P. vivax* patient.
(DOCX)
