# Supplementary material for: Spleen Rupture in a Case of Untreated Plasmodium vivax Infection
Source: PLoS Negl Trop Dis. 2012 Dec 13;6(12):e1934. doi: 10.1371/journal.pntd.0001934 (PMC3521714; doi:10.1371/journal.pntd.0001934)
Supplement: Table S1 — Markers, application, and findings used in the phenotypic in situ characterization and distribution of cells in the spleen of the P. vivax patient. (DOCX) [file pntd.0001934.s001.docx]

**Table S1. Phenotypic in situ characterization of cells in the spleen of the *P. vivax* patient.**

| **MARKER** | **APPLICATION** | **MAIN FINDINGS** |
| --- | --- | --- |
| CD20, CD79a | B-cell lineage | Mild follicular hyperplasia |
| CD10, bcl-6 | Follicular center cells | Mild follicular hyperplasia |
| CD68 KP1 | Monocytes, macrophages and myeloid cells | Expansion monocytes and macrophages |
| CD138 | Plasma cells | Plasma cells and plasmablasts expansion in subcapsular and perivascular areas |
| MUM-1 | Plasma cells and activated T-cells | Plasma cells and plasmablasts expansion in subcapsular and perivascular areas |
| Ki67 | Proliferating cells | Expression in subcapsular and perivascular areas |
| IgD | Delta-chains of human IgD | Main expression in B-cells |
| IgA | Alpha-chains of human IgA | No expression |
| IgM | Mu-chains of human IgM | Main expression in B-cells |
| IgG | Gamma-chain human IgG | Main expression in plasma cells |
| Lambda-light chains | Lambda light chain | Polytipic expression in plasma cells |
| Kappa-light chains | Lambda light chain | Polytipic expression in plasma cells |
| Granzyme B | Cytotoxic granules | No significant changes |
| CD2,CD3,CD5,CD7 | T-cell lineage | Mild cord hyperplasia |
| CD4 | T-helper cells and antigen presenting cells | No significant changes |
| CD8 | T-cytotoxic cells, littoral cells of the spleen | No significant changes |
| CD16 | NK cells, histiocytes | No significant changes |
| CD31 | Endothelial cells | No significant changes |
| CD33 | Myeloid and monocytic cells | No significant changes |
| CD34 | Endothelial cells, stem cells | No significant changes |
| CD56 | Natural killer (NK) cells | No significant changes |
| CD57 | T follicular helper cells | No significant changes |
| CD61 | Megakariocytes | No significant changes |
| CD123 | Plasmocytoid dendritic cells | No significant changes |
| FOX P3 | T-regulatory cells | No significant changes |
| TCR beta | Alpha-beta T-cells | No significant changes |
| Myeloperoxidase | Neutrophil granulocytes and monocytes | No positive cells |
| CD235a, glycophorin A | Normal erythroid cells at all differentiation stages | No positive erythroid precursors |
